# P-2187. Integrated Metabolomic and Single-Cell Transcriptomic Analysis Reveals Neutrophil Heterogeneity and Metabolic Pathways in Severe Hand, Foot, and Mouth Disease Caused by Coxsackievirus A6 Infection

**DOI:** 10.1093/ofid/ofaf695.2350

**Published:** 2026-01-11

**Authors:** Yaping Li, Meng Zhang, Chenrui Liu, huiling Deng, Muqi Wang, Yufeng Zhang, Yuan Chen, Mei Li, Shuangsuo Dang

**Affiliations:** Xi'an Jiaotong University Second Affiliated Hospital, Xi'an, Shaanxi, China; The Second Affiliated Hospital of Xi'an Jiaotong University, Xi'an, Shaanxi, China; Xi'an Jiaotong University Second Affiliated Hospital, Xi'an, Shaanxi, China; Xi'an Children's Hospital, Xi'an, Shaanxi, China; The Second Affiliated Hospital of Xi'an Jiaotong University, Xi'an, Shaanxi, China; Xi'an Children's Hospital, Xi'an, Shaanxi, China; Xi'an Children's Hospital, Xi'an, Shaanxi, China; The Second Affiliated Hospital of Xi'an Jiaotong University, Xi'an, Shaanxi, China; Second Hospital of Xi'an Jiaotong University, Xi'an, Shaanxi, China

## Abstract

**Background:**

Hand, foot, and mouth disease (HFMD) is a significant infectious disease that can lead to neurological damage in children, with Coxsackievirus A6 (CA6) emerging as the predominant pathogen in recent years. We aim to explore the metabolic and transcriptomic profiles of CA6-induced severe HFMD in children.

**Methods:**

Peripheral blood mononuclear cells (PBMCs) and plasma samples were collected from children during the acute and recovery phases of CA6-associated HFMD. Single-cell sequencing and high-throughput targeted metabolomic analysis were conducted, followed by an integrated analysis of the interaction networks.

**Results:**

Single-cell sequencing identified seven distinct cell types in PBMCs. Significant shifts were observed in the proportions of CD4+ naive T cells, neutrophils, CD16- monocytes and naive B cells. Metabolomic analysis of 25 common amino acids revealed significant changes in the glycine, serine, and threonine metabolic pathways. Neutrophils were found to have the highest metabolic scores for glycine, serine, and threonine pathways by scMetabolism. Furthermore, CellChat analysis of the PBMC microenvironment revealed potential molecular interactions between neutrophils and other cell types, including ADGRE5-CD55, ANXA1-FPR1, and CD22-PTPRC. The interaction between neutrophils and these metabolic pathways suggested that FCGR3B and AOC3 may play key roles in mediating these effects.

**Conclusion:**

Alterations in glycine, serine, and threonine metabolic pathways in neutrophils may contribute significantly to the pathogenesis of severe CA6 HFMD.

**Disclosures:**

All Authors: No reported disclosures

